# Residual and Late Onset Symptoms Appeared in a Patient with Severe Fever with Thrombocytopenia in a Convalescence Stage

**DOI:** 10.3390/v13040657

**Published:** 2021-04-10

**Authors:** Kohei Kanda, Noriko Kinoshita, Satoshi Kutsuna, Keiji Nakamura, Ayako Okuhama, Akira Shimomura, Takeshi Inagaki, Tomoki Yoshikawa, Takeshi Kurosu, Masayuki Shimojima, Masayuki Saijo, Norio Ohmagari

**Affiliations:** 1 Disease Control and Prevention Center National Center for Global Health and Medicine Hospital, Tokyo 162-8655, Japan; kkanda@hosp.ncgm.go.jp (K.K.); skutsuna@hosp.ncgm.go.jp (S.K.); keinakamura@hosp.ncgm.go.jp (K.N.); ayokuhama@hosp.ncgm.go.jp (A.O.); ashimomura@hosp.ncgm.go.jp (A.S.); tinagaki@hosp.ncgm.go.jp (T.I.); nohmagari@hosp.ncgm.go.jp (N.O.); 2 Department of Virology I, National Institute of Infectious Diseases, Tokyo 162-8640, Japan; ytomoki@nih.go.jp (T.Y.); kurosu@niid.go.jp (T.K.); shimoji-@nih.go.jp (M.S.); msaijo@nih.go.jp (M.S.)

**Keywords:** severe fever with thrombocytopenia syndrome, convalescence, viral hemorrhagic fevers

## Abstract

Severe fever with thrombocytopenia syndrome (SFTS) is an emerging tick-borne infectious disease caused by *Dabie bandavirus* (formerly SFTS virus, SFTSV). Its manifestations during the convalescent phase have not been widely described. We report a patient presenting with hematospermia, fatigue, myalgia, alopecia, insomnia, and depression during the recovery phase of SFTS. Since these symptoms are widely observed in patients with viral hemorrhagic fevers, there might be common mechanisms between SFTS and other viral hemorrhagic fevers. Close monitoring may be required during the recovery phase of SFTS.

## 1. Introduction

Severe fever with thrombocytopenia syndrome (SFTS) is an emerging tick-borne infectious disease caused by *Dabie bandavirus* (formerly SFTS virus, SFTSV) belonging to the *Bandavirus* genus (formerly *Phlebovirus* genus) of the *Phenuiviridae* family (formerly *Bunyaviridae* family), which should be categorized as a viral hemorrhagic fever disease [1]. The acute symptoms and pathophysiology of the disease are not fully understood and require further investigation [1]. Moreover, the manifestations that occur during the convalescent phase have not been widely described. Here, we report a patient presenting with hematospermia, fatigue, myalgia, alopecia, insomnia, and depression during the recovery phase of SFTS.

## 2. Case Report

**Figure 1:**
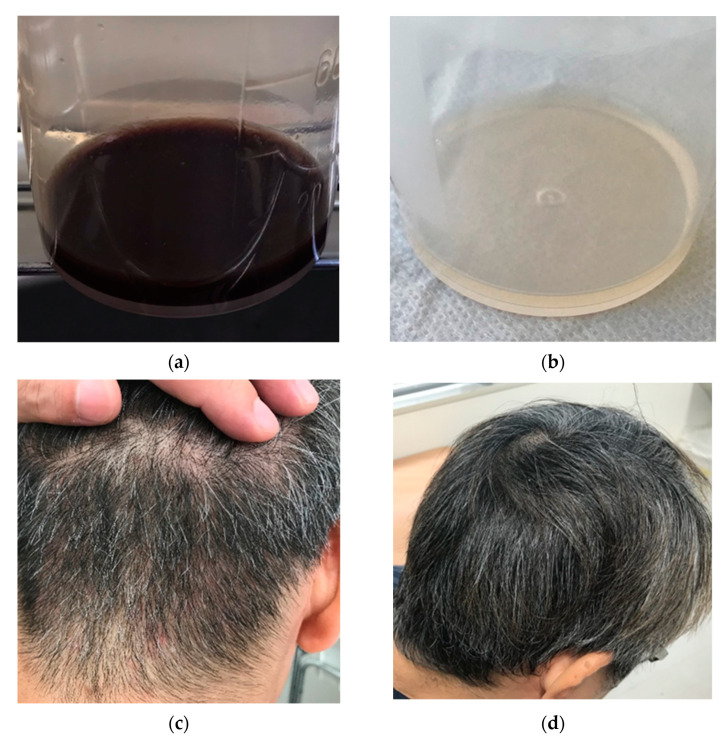
Visually observed symptoms of (**a**) semen samples with gross hematospermia obtained on Day 26, (**b**) semen, in which gross hematospermia had disappeared, obtained on Day 32, (**c**) diffuse alopecia recorded on Day 86, and (**d**) the hair condition, in which alopecia had resolved, recorded on Day 112.

**Figure 2:**
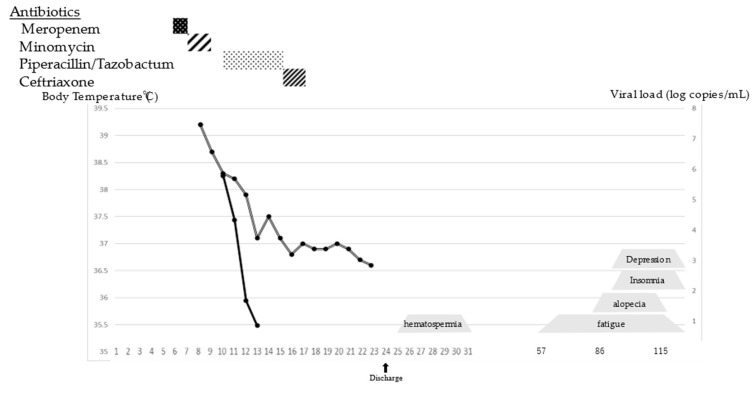
Timeline for the patient’s symptoms, body temperature(double line), viral load(solid line), and administration of broad-spectrum antibiotics.

The patient was a 53-year-old man living in Tokyo. He had traveled to Goto City, Nagasaki Prefecture, Western Japan, which is an SFTS-endemic area, 6 to 1 days before the onset. A cat was kept in the accommodation; however, the patient did not have direct contact with it or any other mammals. A day before his return to Tokyo, he developed anorexia and a headache. After returning to Tokyo, he complained of fever, vomiting, and diarrhea. On Day 4 of symptom onset, he visited a family doctor and underwent a blood test, which revealed leukocytopenia and thrombocytopenia. He was subsequently prescribed symptomatic drugs. His symptoms did not improve and he experienced an altered state of consciousness on Day 7. He was admitted to the emergency department of a hospital, and a total blood cell (TBC) test revealed a white blood cell count of 1.2 × 10^3^/µL (normal range: 3.3–8.6 × 10^3^/µL) and a platelet count of 5.2 × 10^3^/µL (normal range: 158–348 × 10^3^/µL). There was a marked increase in the creatinine kinase level (14,853 U/L; normal range: 41–153 U/L) and a mild increase in the C-reactive protein (CRP) level (0.73 mg/dL; normal range: 0.00–0.014 mg/dL). For suspected sepsis, he was administered broad-spectrum antibiotics. On Day 8, bone marrow aspiration was performed, which showed the features of atypical lymphocytosis but no evidence of hemophagocytosis. He was transferred to the National Center for Global Health and Medicine (NCGM) on the same day for further examination. On initial examination at the NCGM, his temperature was 38.9 °C and he had no abnormal findings except congested bulbar conjunctiva. No visible tick bites were identified. A TBC test revealed persistent leukocytopenia and thrombocytopenia (Table 1). The patient was suspected of having SFTS due to his travel history and blood test findings. Blood and urine samples were tested for SFTSV RNA through SFTSV genome detection with quantitative real-time reverse transcription-polymerase chain reaction (qRT-PCR) [2], and the SFTSV genome (J1 genotype) was detected [3]; thus, the patient was diagnosed as having SFTS. The viral load was 1.76 × 10^6^ copies/mL and 1.90 × 10^2^ copies/mL in the serum and urine, respectively. With supportive care, he regained consciousness on Day 13, and the hematological and biochemical abnormalities began to improve. A blood sample on Day 19 was also tested for qRT-PCR, showing a negative reaction. The patient was discharged on Day 24. On the first outpatient follow-up on Day 33, the patient complained of hematospermia (Figure 1A). Semen obtained on Day 26 was tested for the presence of the SFTSV using qRT-PCR [2]; however, a negative result was obtained. Urine analysis and serum prostate-specific antigen levels were normal. The gross hematospermia was resolved by the next visit on Day 39 (Figure 1B). On Day 80, the patient presented with fatigue, myalgia of the lower legs, diffuse alopecia (Figure 1C), insomnia, and depression. His hair was easily removed by traction, but other physical findings and blood tests showed no abnormalities. At the latest follow up on Day 536, hair loss was improved but chronic fatigue and depression persisted (Figure 2).

## 3. Discussion

To the best of our knowledge, SFTS has not been previously reported in Eastern Japan. At present, ~500 cases of SFTS have been reported in Japan and all were from Western Japan, except for the patient in the present report [4]. This suggests that SFTS cases can be encountered in non-endemic areas, particularly in cases with a travel history to an endemic area.

The pathophysiology of SFTS and the symptoms occurring in the acute phase are not fully understood [5]. Moreover, the manifestations occurring in the recovery period are less well known. The patient in the present report complained of hematospermia, fatigue, myalgia, diffuse alopecia, insomnia, and depression during the recovery period, some of which have been previously known in some viral hemorrhagic fevers. For instance, survivors of the Ebola virus disease report a range of sequelae, described as the post-Ebola syndrome, which is characterized by musculoskeletal pain, headache, and ocular problems [6,7]. In other viral hemorrhagic fevers, labile pulse, tachycardia, temporary complete loss of hair, polyneuritis, difficulty in breathing, xerostomia, poor vision, loss of hearing, and loss of memory in patients with Crimean-Congo hemorrhagic fever; hearing loss or impairment with Lassa fever; and asthenia, myalgia, fever, orchitis, and uveitis with Marburg hemorrhagic fever are also known [8,9,10,11,12]. Although some of these complications are commonly found in various critical illness other than viral hemorrhagic fevers [13,14], SFTS is an infectious disease that is included in the viral hemorrhagic fevers, and there might be common pathophysiological mechanisms behind the complications in the recovery phase among viral hemorrhagic fevers. One hypothesis is that chemokines and cytokines such as interleukin (IL)-1β, IL-12p40, IL-17, and vascular endothelial growth factor, which have been found to be elevated during the recovery phase of SFTS [15], cause the convalescent phase symptoms. In the present case, we did not measure these cytokines; thus, the actual relationship between the symptoms of the present case and cytokines is unclear. Further studies are required to clarify the mechanisms causing these convalescent phase symptoms in patients with SFTS. In addition, the long-term impact of these symptoms on the patients’ quality of life or prognosis is not clearly understood. Further accumulation of cases of various severities is required to estimate the overall impact of the disease.

Furthermore, the symptoms of this patient may have implications for protection against SFTSV in certain situations. Hematospermia has been reported in a patient with Zika virus disease as well, which can be transmitted through sexual intercourse [16]. Although person-to-person transmission of the SFTSV through bodily fluids other than semen have been identified [17,18] and SFTSV RNA has been detected in semen [19], there have been no reports on patients with SFTSV who were sexually transmitted with SFTSV. The risk of sexual transmission remains unclear.

## 4. Conclusions

We reported a case of a patient with SFTS in Tokyo, Japan, an area in which the disease has not been previously reported. The patient exhibited hematospermia, diffuse alopecia, fatigue, and depression during the convalescent phase. Close patient monitoring may be required during the recovery phase of SFTS, especially in severe cases, to improve the quality of life for those recovered from SFTS, one of the severe viral hemorrhagic fevers with high morbidity and mortality.

## Figures and Tables

**Table 1:** Blood test result on admission (Day 8).

Test	Value	Reference Range
Leukocyte count	1200 /µL	3300–8600 /µL
%Neutrocyte	61.8 %	
%Lymphocyte	34.4 %	
Hemoglobin	19.4 g/dL	11.6–14.8 g/dL
Thrombocyte	52,000 /µL	158,000–348,000 /µL
Sodium	134 mEq/L	138–145 mEq/L
Potassium	3.5 mEq/L	3.6–4.8 mEq/L
Chloride	97 mEq/L	101–108 mEq/L
Blood urea nitrogen (BUN)	44.0 mg/dL	8.0–22.0 mg/dL
Creatinine	1.97 mg/dL	0.46–0.79 mg/dL
Glucose	121 mg/dL	70–109 mg/dL
Total bilirubin	0.8 mg/dL	0.4–1.5 mg/dL
Albumin	3.6 g/dL	3.9–5.3 g/dL
Alkaline phosphatase	353 U/L	106–332 U/L
Aspartate aminotransferase	1019 U/L	13–30 U/L
Alanine aminotransferase	254 U/L	7–23 U/L
Gamma-glutamyl transpeptidase	56 U/L	10–80 U/L
Creatinine kinase	14,853 U/L	57–197 U/L
Lactate dehydrogenase	480 U/L	124–222 U/L
Prothrombin time-international normalized ratio	1.09	0.90–1.10
**Activated partial thromboplastin time**	**39.0 s**	**25.0–40.0 s**
**Fibrinogen**	**278 mg/dL**	**200.0–400.0 mg/dL**
**D-dimer**	**94.1 µg/mL**	**0.00–1.00 µg/mL**

## Data Availability

Not applicable.
